# The Management of a Geriatric Patient Using Dabigatran Therapy on Dentigerous Cyst with Oral Bleeding

**DOI:** 10.3390/jcm13051499

**Published:** 2024-03-05

**Authors:** Glauco Chisci, Dafne Chisci, Enea Chisci, Viola Chisci, Michela Stumpo, Elettra Chisci

**Affiliations:** 1 Centro Dentistico Chisci, Via Ricasoli 18, 58100 Grosseto, Italy; chiscidafne@gmail.com (D.C.); elettra.chi@gmail.com (E.C.); 2 Department of Ophthalmology, University of Palermo, 90133 Palermo, Italy; enea.chisci@gmail.com; 3 Department of Ophthalmology, Greifswald University Hospital, 17475 Greifswald, Germany; 4 Department of Pathology, USL Toscana Sudest—Grosseto, Misericordia Hospital, 58100 Grosseto, Italy

**Keywords:** jaw cyst, bleeding, cyst, neoformation, dabigatran, elderly, dentigerous cyst, follicular cyst, third molar, impacted third molar, inferior alveolar nerve, mandibular nerve

## Abstract

Jaw cysts represent a great matter of interest in oral and maxillofacial surgery. Jaw cyst surgery is a common intervention in oral surgery but, in the case of a multidisciplinary patient, the oral surgeon needs to meet with other specialists. A cyst is an epithelium-lined sac containing fluid and/or semisolid material due to epithelial cell proliferation, degeneration, and liquefaction; the hypertonic solution withdraws liquids from the surrounding tissues, while internal pressure exerts an equal strength on the cyst walls. Dentigerous cysts are the second most common odontogenic cysts after radicular cysts, and commonly few or no symptoms are reported. However, the most common diagnosis for dentigerous cyst is represented by eruption of the affected tooth or accidental diagnosis. Commonly, dentigerous cysts may be related to impacted third molars; in the case of impacted third molars and a dentigerous cyst, the tooth should be removed along with the cyst in the same intervention. Mandibular dentigerous cysts are common in children and adults, while dentigerous cysts are a rare neoformation in elderly patients. Treatment usually involves removal of the entire cyst and the associated unerupted tooth. This intervention may be more difficult if the cyst is large, the third molar is in contact with the mandibular nerve, and/or the patient has a medical history that may represent a relative or absolute contraindication. We present the case of a rare symptomatic manifestation of dentigerous cyst in an elderly patient in treatment with dabigatran therapy; for the treatment of dentigerous cysts in the elderly, we suggest a multidisciplinary approach with the use of the histological examination and a careful follow-up.

## 1. Introduction

Jaw cysts represent a great matter of interest in oral and maxillofacial surgery. Jaw cyst surgery is a common intervention in oral surgery and, on the basis of diagnosis, the prognosis may vary [1]. A cyst is an epithelium-lined sac containing fluid and/or semisolid material due to epithelial cell proliferation, degeneration, and liquefaction; the hypertonic solution withdraws liquids from the surrounding tissues, while internal pressure exerts an equal strength on the cyst walls [2]. Dentigerous cysts are the second most common odontogenic cysts after radicular cysts [3]. The most common diagnosis for a dentigerous cyst is evoked by eruption of the affected tooth or accidental diagnosis. The tooth related to the dentigerous cyst is always an impacted tooth [3]. Typically, dentigerous cysts are painless and are discovered during a routine radiographic examination. The possibility to distinguish a dentigerous cyst from other cyst is a great matter of interest. Complete diagnosis is histological, as the presence of a cyst lining the epithelium of the cubic or columnar type with areas of mucinous metaplasia with the presence of a nest of residues of odontogenic epithelium confirms the diagnosis of a dentigerous cyst [4]. Clinically, dentigerous cysts are related to impacted teeth, and diagnosis is poor. From a radiological point of view, the presence of radiolucency related to an impacted tooth may lead to many differential diagnoses, and the use of cone beam computed tomography may be superior to panoramic radiograph [5].

Over 75% of all cases are located in the mandible, and the third molar is the most commonly associated tooth [6,7]. The third molar pathology represents a common operation in oral and maxillofacial surgery. Although there may be many indications for the extraction of the mandibular third molar, such as orthodontic indication or contact with jaw cyst, the most common cause of third molar extraction remains pericoronaritis, as an infection and inflammation of the pericoronal tissues around the third molar [8]. This is the most frequent pathology of the third molar with dysodontiasis. Dysodontiasis regards alterations related to tooth inclusion, a lack of dragging competence of periodontal ligament, and probably eruptive deficit during dental root development [8]. Pericoronitis, on the other hand, is a typical inflammatory pathology of the impacted third molar that influences the quality of life of the patients before the extraction of the tooth, more commonly occurring at the ages of 20s and 30s [8,9].

The most common symptom of third molar pathology is pain. A characteristic of this pathology is the difficulty of the patient to refer to the upper or lower tooth, as trigeminal innervation often confuses the patient due to the maxillary and mandibular branches. Dysodontiasis and impaction of the third molars are a common condition for most of patients, as some third molars remain impacted for most of a patient’s life and do not cause any symptoms [8,9,10,11,12]. For this reason, the diagnosis of a jaw cyst related to an impacted third molar may be belated, and the surgery is performed on a growth neoformation [13]. Treatment usually involves removal of the entire cyst and the associated unerupted tooth. This intervention may be more difficult if the cyst is large, the third molar is in contact with the mandibular nerve, and/or the patient has a medical history that may represent a relative or absolute contraindication. The performance of surgery on large jaw cysts may include the management of possible mandibular intraoperative fracture and use of a titanium plate for intraoperative fixation [14].

Last but not least, another important aspect is the age of the patient. While the dental management of an elderly or geriatric patient certainly represents a routine difficulty in hygiene and prevention procedures, the management of a surgical intervention raises some different difficulties. In the case of surgery for a dentigerous cyst in a geriatric patient, some medical, anesthetic, and surgical aspects must be coordinated, and a multidisciplinary approach is mandatory. This operation in geriatric patients requires careful consideration due to the potential health risks and the impact of aging on the body. Dentigerous cysts are generally associated with impacted teeth, and in geriatric patients, the approach must be tailored to their specific needs.

The surgical procedure involves removing the cyst and the affected teeth, as in adult and childhood patients, but may require more delicate handling in older patients. Preoperative assessment is crucial to evaluate the patient’s overall health, identify any coexisting medical conditions, and determine the most suitable anesthesia option. In geriatric patients, there may be concerns related to healing, postoperative pain management, and potential complications.

Additionally, collaboration between the oral surgeon, geriatric specialist, and anesthesiologist can contribute to a comprehensive and individualized treatment approach. This interprofessional coordination helps ensure that the surgical procedure is conducted with a patient’s overall well-being in mind.

The pharmacological therapies that mainly influence dental surgery are largely represented by immunosuppressants and chemotherapy for the risk of postoperative infection, bisphosphonates for the risk of postoperative osteonecrosis, psychiatric drugs for the risk of interactions and clearance of postoperative drugs, and anticoagulants for the risk of intraoperative and postoperative bleeding [15,16,17]. Furthermore, patient and caregiver education are important components of the treatment process, as they play a significant role in postoperative care and monitoring for any signs of complications. Overall, the surgical management of dentigerous cysts in geriatric patients necessitates a multidisciplinary approach, meticulous planning, and personalized care to optimize outcomes and minimize potential risks.

With regard to the intake of direct oral anticoagulants in a patient with a dentigerous cyst, this condition makes the surgical management of the clinical case more difficult [18,19]. The intake of an oral anticoagulant may lead to complications after dentoalveolar surgery, and bleeding from any part of the mouth by patients on dabigatran can be spontaneous or triggered by trauma or pressure. Incidental diagnosis of a dentigerous cyst after bleeding is uncommon. Although an experienced oral surgeon treats dentigerous cysts with ease, the same cannot be said for a cardiologist, a primary care physician, or a geriatrician, who are common readers for the present journal.

Dabigatran etexilate is an anticoagulant drug belonging to the new category of direct thrombin inhibitors, which are part of the group called new oral anticoagulants [20].

These new oral anticoagulants demonstrate a dramatic reduction in the rate of intracranial hemorrhage as compared to warfarin and offer the advantages of absolution of monitoring, therefore averting the risk of hemorrhage in the context of a narrow therapeutic window, as well as the under-treatment characteristic of warfarin, particularly in high-risk patients. One major concern and disadvantage of these drugs was the lack of reversal agents, which has largely been ameliorated by the approval of idarucizumab for dabigatran and andexanet alfa for both apixaban and rivaroxaban, with ciraparantag as a universal reversal agent for all new oral anticoagulants undergoing fast-track review from FDA [21]. Balancing the risks of bleeding and thrombosis after acute myocardial infarction is challenging; additionally, the use of dabigatran in these patients may reduce the rate of all-cause mortality and may have little or no effect on cardiovascular mortality [22].

In the present article, we report the clinical case of a rare symptomatic manifestation of a dentigerous cyst in an elderly patient in treatment with dabigatran therapy and discuss multidisciplinary approach that was used, along with histological examination; further, we perform a literature review in order to investigate previous treatments and compare the patient data of previous cases and the present case.

## 2. Case Report

**Figure 1:**
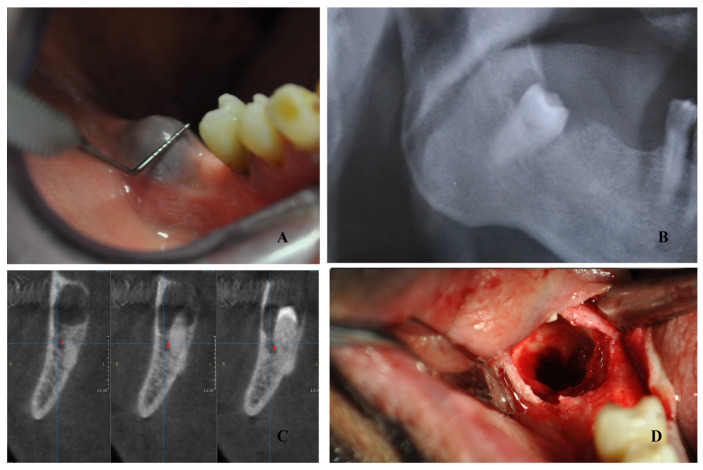
A rounded swelling area in the right mandibular third molar site (**A**). Panoramic radiograph image with impacted mandibular right third molar surrounded by a radiolucent lesion (**B**). CBCT reveals alveolar nerve involvement (**C**). Intraoperative image after cyst and third molar removal (**D**).

An 81-year-old patient was urgently referred to the first author for oral bleeding and swelling. The patient reported ischemic heart disease and had therefore been treated with anticoagulant oral therapy(dabigatran) for many years. The patient took dabigatran once daily, in the morning. At the emergency visit, during the oral examination, a rounded swelling area was detected in the right mandibular third molar site, with a soft consistency and the presence of small clots in the oral cavity (Figure 1). The patient referred to spontaneous bleeding from this area, and the increasing size of this mass made the removable prosthesis of the patient uncomfortable to wear. Panoramic radiograph revealed an impacted mandibular right third molar surrounded by a radiolucent lesion (Figure 1). Tranexanic acid mouth rinses and gauze applications were prescribed as immediate therapy. Cone beam computed tomography (CBCT) was then performed, and the contact between the jaw cyst with the third molar and inferior alveolar nerve was confirmed (Figure 1).

The decision to the correct operational treatment plan involved the patient’s reference cardiologist and geriatrician doctor. After evaluating the clotting profile with INR, PT, PTTK, and complete blood count, a suspension of dabigatran surgery was decided on morning of the surgery and lasted for 24 h. The new schedule was started the following day, with an accurate hemostasis and hybrid follow-up (serial telephone checks every 3 h the day after the extraction with repeated oral instructions and outpatient visits at two and seven days after extraction) to monitor the bleeding. The reference anatomopathologist of the facility was consulted for follow-up after the surgery.

**Figure 2:**
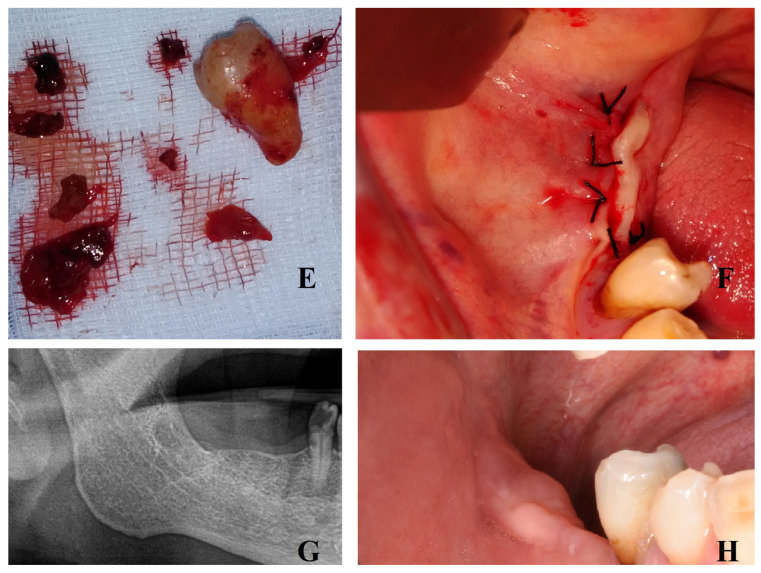
Tooth and cystic sample (**E**). Mattress sutures (**F**). Panoramic radiograph 1 year after surgery shows complete recovery (**G**). Clinical image of recovery (**H**).

A complete oral hygiene was performed 7 days before surgery, and 0.12% chlorexidine rinses were prescribed for 7 days before surgery. The intervention was performed under local anesthesia: mandibular block with articaine 1:200,000. Afterwards, a crestal incision was performed over the third molar site without a releasing incision; a full-thickness periosteal flap was then elevated to display the mass. Once the cyst was observed, the cortical bone was removed with a side-cutting rongeur plier and the cystic epithelium was gently dislodged. Afterwards, the third molar was gently luxated and extracted, and the residual cyst epithelium was removed (Figure 1). Cystic tissue was collected for histological examination. A complete cleaning of the alveolar pocket was carried out with hydrogen peroxide. Afterwards, sutures were applied with 4/0 mattress stitches (Figure 2).

## 3. Follow-Up

**Figure 3:**
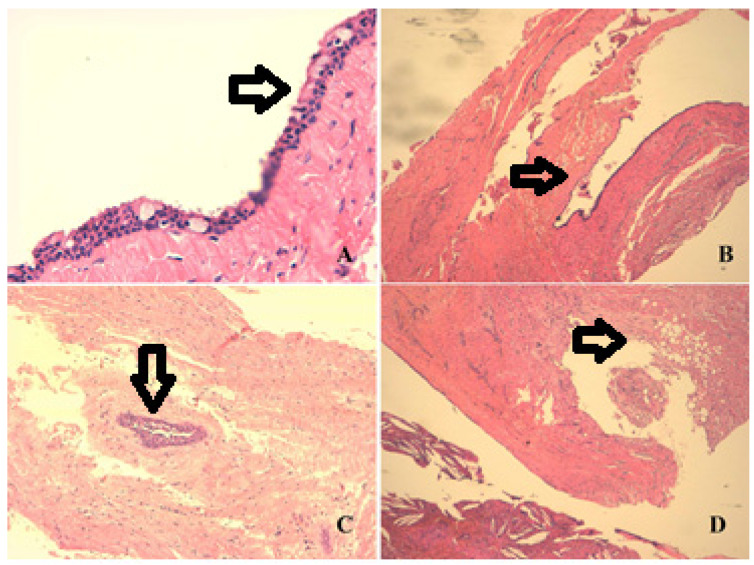
Arrows represent the description. Histological image of the dentigerous cyst: we may appreciate the detail of mucinous metaplasia, typical of a dentigerous cyst (**A**). Cyst lining the epithelium of the cubic/columnar type, with areas of mucinous metaplasia (**B**). Nest of residues of odontogenic epithelium that confirm the diagnosis of a dentigerous cyst (**C**). Myxoid aspect of the cyst wall (greyish area) and the cholesterol crystals also present in the lower part of the image with the chronic giant cell inflammation (**D**).

No complications occurred, in particular no excessive bleeding, neither during the surgery nor in the week after. Mandibular sensitivity returned with the pass of the dental anesthesia. Seven days after surgery, the sutures were removed. Initial histologic examination revealed a dentigerous cyst (Figure 3). One year after surgery, a new panoramic radiograph was performed, revealing the complete healing of the site (Figure 2).

## 4. Discussion

This paper reports the management of a symptomatic dentigerous cyst associated with an impacted third molar in an elderly patient. Few cases of symptomatic dentigerous cysts in elderly people are reported in the literature. Further, in the case of inferior alveolar nerve impairment due to third molar surgery, a follow-up with lip sensibility evaluation is mandatory [23]. The removal of oral and maxillofacial neoformation is a common matter in outpatient surgery, and it requires a correct knowledge of anatomy and hemostasis procedures. Histological examination is mandatory in order to set up the correct follow-up.

Bleeding is more likely to result from repetitive pressure or trauma on the overlying oral mucosa from the denture and is least likely to be from the cyst. The differential diagnosis considered a hemorrhagic eruptive cyst, but we considered this theory unlikely due to the age of the patient. The histological examination reported in Figure 3 confirmed the nature of the dentigerous cyst.

Dentigerous cysts are usually non-symptomatic but, in this case, the clinical manifestation was the determinant in revealing the nature of this pathology. Few papers in the literature have reported significant data regarding dentigerous cyst management in geriatric patients. Nishide et al. [24] have reported good results in terms of bone regeneration with irrigation therapy instead of cyst removal. In the present case, the association of the dentigerous cyst with recurrent bleeding did not let us undertake this treatment, as leaving the dentigerous cyst without treatment could lead to recurrent bleeding. Loomba et al. [25] have, in their case, which is similar to the present case, reported the extraction of an impacted premolar and cyst enucleation in a 72-year-old patient referred for swelling. On the basis of our experience, we decided to use antibiotic prophylaxis and a hybrid postoperative follow-up to evaluate any early complications; this information was not present in the previous study.

Bleeding does not appear as an incidental finding in the international literature, nor as a referral symptom; meanwhile, in the present case, oral bleeding resulted in being the significant factor for the investigation of the dentigerious cyst. In Table 1, anagraphic data and a comparison of the international literature with the present case are reported. The main symptoms of referral by the patient in this study was bleeding and intraoral swelling, which are uncommon symptoms for a dentigerous cyst. Spontaneous bleeding in elderly patients with dabigatran therapy is a rare event and requires a multidisciplinary approach [26,27,28].

Since their introduction, direct oral anticoagulants have changed the landscape of the therapeutic treatment of venous thromboembolism and atrial fibrillation. These drugs represent standard treatments, and cases of spontaneous bleeding are uncommon. The main dreaded hemorrhages are intracranial or gastrointestinal [29]. Intraocular bleeding, even if not a fatal hemorrhage, is reported as a dangerous collateral effect for the visual loss it causes [30]. However, oral bleeding with dabigatran therapy is not reported in the international literature; this aspect could be due to its possible inclusion in a more general gastrointestinal hemorrhage, or it may be an under-evaluated event compared with the worst collateral effects previously reported. For a dentigerous cyst, the event of spontaneous oral bleeding is even less common, and the importance of this case report is underlined by the rarity of this sum of rare conditions: oral spontaneous bleeding with direct anticoagulants and a symptomatic dentigerous cyst in an elderly patient. A dentigerous cyst in a geriatric and medically compromised patient is a rare case and demands special care and attention; furthermore, the decision to cease anticoagulant before surgery or not is of critical importance. The removable prosthesis of this patient could have played a role too: the compression of the prosthesis over the impacted tooth and cyst could evoke a bleeding. However, the patient referred to having worn the prosthesis for many years.

With regard to oral surgery procedures that have been performed to maintain hemostasis, a recent literature review evaluated first or secondary closures of third molar surgeries; unfortunately, the studies of third molar surgery do not include the jaw cyst pathologies [31]. For this case, due to the great cavity left by the operation and the bleeding risk, we considered the usefulness of mattress stitches to close the wound by primary intention and reduce the bleeding. The sutures were removed seven days after surgery. With regard to the postoperative recovery after third molar surgery, the main attention after bleeding was on surgical site infection and alveolar osteitis [32]. The literature suggests the use of platelet-rich fibrin and/or ozone in order to reduce this risk [33,34]. The scientific research on oral surgery has studied several advanced hemostasis methods. With regard to anticoagulant treatments in patients with dentigerous cysts, Carter et al. [35] have reported good results for hemostasis with autologous fibrin glue. Kwon et al. [36] have reported a rare case of hemophilic pseudotumor in the maxilla and have suggested, in case of jaw neoformations in hemophilic patients or patients taking anticoagulants, to consider cases for differential diagnosis. This aspect is very interesting, and it underlines the role of the histological examination that we performed in the present case. The complete diagnosis of a jaw cyst is very important. Wong et al. [37] have reported a geriatric case affected by glandular odontogenic cyst that received surgery in order to restore occlusion with a removable prosthesis. Recently, scientific interest in the study of oral neoformations has grown. Lei et al. [38] have documented an interesting report on oral neoformations in the older Taiwanese population, suggesting that more research be performed on this matter.

This paper reports the first case of spontaneous oral bleeding as the first clinical sign of a dentigerous cyst in a geriatric patient with one year of follow-up; while the one year of follow-up may appear the limit, a literature review was performed to compare the characteristics of the previous two cases reported of dentigerous cysts in geriatric patients. Clinicians should consider the possibility of dentigerous cysts in elderly patients and, on the basis of a multidisciplinary evaluation with a cardiologist and geriatrician doctor, choose the most effective treatment to reduce the risks of surgery.

## 5. Conclusions

The management of jaw cysts and the third molar associated with anticoagulant oral therapy in elderly patients is a matter of interest for cardiologists and geriatrician doctors. Care is mandatory in patients with a complicated medical history, and a comprehensive multidisciplinary evaluation is needed. Histological examination is the key to the correct diagnosis of jaw cysts, and postoperative follow-up in the mid and long term plays an important role. With the present case’s presentation, we report our experience and suggest a tailored care approach and experience in oral surgery for geriatric patients.

## Figures and Tables

**Table 1:** Comparison data of literature review regarding dentigerous cysts in geriatric patients.

Authors	Year	Age	Sex	Chief Complaint	Drugs	Pathology	Treatment	Reference
Nishide et al.	2003	72	male	No symptoms		Dentigerous cyst	Irrigation	[24]
Loomba et al.	2011	72	male	Swelling	Aspirin	Dentigerous cyst related to impacted lower premolar	Cyst enucleation and tooth extraction	[25]
Chisci et al.	2024	81	Male	Bleeding	Dabigatran	Dentigerous cyst related to impacted lower third molar	Cyst enucleation and tooth extraction	-
